# Multiheaded Cationic Surfactants with Dedicated Functionalities: Design, Synthetic Strategies, Self-Assembly and Performance

**DOI:** 10.3390/molecules28155806

**Published:** 2023-08-01

**Authors:** Łukasz Lamch, Weronika Szczęsna, Sebastian J. Balicki, Marcin Bartman, Liliana Szyk-Warszyńska, Piotr Warszyński, Kazimiera A. Wilk

**Affiliations:** 1 Department of Engineering and Technology of Chemical Processes, Wrocław University of Science and Technology, Wybrzeże Wyspiańskiego 27, 50-370 Wrocław, Poland; lukasz.lamch@pwr.edu.pl (Ł.L.); weronika.szczesna@pwr.edu.pl (W.S.); sebastian.balicki@pwr.edu.pl (S.J.B.); marcin.bartman@pwr.edu.pl (M.B.); 2 Jerzy Haber Institute of Catalysis and Surface Chemistry, Polish Academy of Sciences, Niezapominajek 8, 30-239 Kraków, Poland; lilianna.szyk-warszynska@ikifp.edu.pl (L.S.-W.); piotr.warszynski@ikifp.edu.pl (P.W.)

**Keywords:** multifunctional surfactants, custom-designed structures, synthetic methodologies, properties at interfaces and solution, high performance

## Abstract

Contemporary research concerning surfactant science and technology comprises a variety of requirements relating to the design of surfactant structures with widely varying architectures to achieve physicochemical properties and dedicated functionality. Such approaches are necessary to make them applicable to modern technologies, such as nanostructure engineering, surface structurization or fine chemicals, e.g., magnetic surfactants, biocidal agents, capping and stabilizing reagents or reactive agents at interfaces. Even slight modifications of a surfactant’s molecular structure with respect to the conventional single-head–single-tail design allow for various custom-designed products. Among them, multicharge structures are the most intriguing. Their preparation requires specific synthetic routes that enable both main amphiphilic compound synthesis using appropriate step-by-step reaction strategies or coupling approaches as well as further derivatization toward specific features such as magnetic properties. Some of the most challenging aspects of multicharge cationic surfactants relate to their use at different interfaces for stable nanostructures formation, applying capping effects or complexation with polyelectrolytes. Multiheaded cationic surfactants exhibit strong antimicrobial and antiviral activity, allowing them to be implemented in various biomedical fields, especially biofilm prevention and eradication. Therefore, recent advances in synthetic strategies for multiheaded cationic surfactants, their self-aggregation and performance are scrutinized in this up-to-date review, emphasizing their applications in different fields such as building blocks in nanostructure engineering and their use as fine chemicals.

## 1. Introduction

**Scheme 1:**
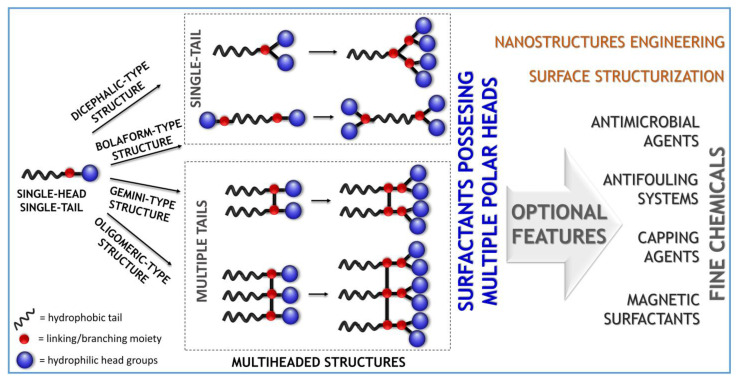
The general idea for customizing surfactants possessing multiple charge heads.

Nowadays, surfactants constitute one of the major groups of chemical compounds, and they are of fundamental importance to household and cosmetics chemistry, petrochemical and pharmaceutical industries, the high-tech sector and nanomedicine, as well as polymeric additives, adhesives and paints. They are being continuously designed to attain products with specific physicochemical properties for targeted applications [1,2,3,4,5]. Physicochemical and biological properties, as well as the performance of present-day surface-active compounds, so-called custom-designed (or customized), multifunctional or value-added surfactants, may be controlled via certain slight structural modifications: functionalizing engineered surfactant structures via the incorporation of an additional alkyl chain (e.g., to obtain double-tail single-head structures) or headgroup (e.g., to obtain multicharge or multiheaded structures), as well as by varying the linker, connecting the hydrophobic and hydrophilic parts; mainly, its type and chemical reactivity [3,6,7,8,9]. There are many possible linkages, such as esters, disulfides, amides, acetals, ketals and azo groups (amongst others), which can be placed between the hydrophobic and hydrophilic portions of the surfactants, categorized in terms of sensitivity both to changes in pH, CO_2_ levels, light, magnetic field or electrical potential and to added enzymes or chemically labile systems that undergo bond cleavage [3,9,10,11,12]. A schematic depiction of optional features achieved via the customization of multiheaded surfactants, further demonstrating that the molecular architecture can result in interesting functional properties, is shown in Scheme 1.

Multiheaded surfactants can be classified into several categories; for example, dicephalic surfactants containing a single tail and two hydrophilic head groups [1,13], gemini (twin or dimeric) surfactants in which two “heads” and two “tails” are separated by a spacer [14,15]; bolaform surfactants containing two hydrophilic groups located at opposite ends of the hydrophobic chain [16]; and oligomeric structures, including trimeric representatives, that have three or more number of heads (generally called as multiple) attached to multiplied hydrophobic fragments [17,18,19]. Oligomeric-type structures generally comprise more than two amphipatic entities covalently linked by an appropriate spacer at or in the headgroups’ vicinity, and the spacer can be linear, star-shaped or ring-like in design [20]. Finally, there are also dendrimeric surfactants (so-called dendritic ones) having dendronium-type heads, which are formed by covalently linking a hydrophobic tail and a hydrophilic dendritic unit—a bulky multicharge head [21].

Dendronic head groups possess very different structural features than simple surfactant head groups, and their self-assembly behavior differs from that of conventional surfactants. They reveal special aggregation behavior in the solution and at the interfaces, along with some important advantages such as a controllable molecular structure, geometric symmetry of molecular configuration, variable types and a number of functional groups [22]. In the literature, there are some reports on the dendrimeric polymer amphiphiles (often so-called dendritic polymeric surfactants), their synthesis, aggregation and application properties [23]. However, they constitute derivatives with relatively high polydispersity, whereas dendrimeric surfactants (see Scheme 1), products of low molecular weight, have fixed molecular mass, high solubility and easily controllable aggregation behavior [21,22]. The branched and multiheaded surfactants (Scheme 1: bicephalic, gemini, trimeric representatives) can be considered initial generations of linear dendritic surfactants. Gemini surfactants have branched structures in their headgroups or hydrophobic tails, leading to many unique properties that are superior to those of the single-head–single-tail ones [4]. Wang et al. synthesized a group of surfactants with multiple headgroups by grafting a dendritic unit of poly(amidoamine) onto an octadecyl chain, which formed vesicles in aqueous solutions at pH > 5 [24]. Recently, two generations of carboxylate surfactants C18-G1-(COONa)_2_ and C18-G2-(COONa)_4_ have been synthesized and characterized by Lou et al. [22]. However, the literature rarely describes linear dendritic structures with a strictly symmetric configuration. Very popular lysine amphiphilic dendrimers, first synthesized in the early 1980s, were applied in many biomedical applications as drug or gene nanocarriers as well as antibacterial, antiviral and anti-amyloid agents [21,25].

The present review considers the following issues: (i) strategies for designing new cationic multiheaded surfactant structures and the most suitable synthetic methodologies to achieve them in good yields and purity; (ii) the main features of aggregation phenomena at interfaces and in solution; (iii) new surfactant-templated or surfactant-stabilized functional nanostructures; (iv) desired performance of surfactant–polyelectrolyte complexes in drug delivery; as well as (v) the functional properties of multi-cationic surfactants as fine chemicals. This review provides guidance for the design and fabrication of future new multicharge surfactants with dedicated functionality for evaluating their usefulness and broadening our knowledge about functional materials for various applications in industrial and academic fields.

## 2. Synthetic Approaches

The synthesis of multifunctional surfactants involves multiple approaches combining different chemical reactions. Typically, the most common reactions include any type of substitution, especially that resulting in the formation of novel carbon–carbon or carbon–heteroatom bonds, or addition, e.g., Michael addition or so-called “click” chemistry reactions. The latter approaches are more preferred for synthetic routes due to a lack of any low-molecular-weight byproducts, which may be difficult to separate from the intended product or semiproduct. Detailed information about the particular synthetic routes and approaches is presented in Section 2.1 (for modular-type synthesis), Section 2.2 and Section 2.3 (for coupling reactions). It should be noted that the presented synthetic routes may be utilized not only for the synthesis of multiheaded cationic surfactants but also for any type of multifunctional derivatives of anionic, cationic, zwitterionic or nonionic origin [4,5,26,27,28,29]. Moreover, the general considerations concerning synthetic routes and the preparation of relatively simply multifunctional surfactants (e.g., gemini, biscephalous or bolaform-type ones) are presented below. In addition to the reaction conditions that enable obtaining intended structures without their chemical degradation, the most crucial aspect is the appropriate synthesis strategy involving the design of the structure with the desired features and the optimal preparation route. The design needs to take into consideration several issues such as: (i) the overall hydrophilicity and hydrophobicity of the amphiphilic compound, especially the number and length of alkyl chain lengths (too short, especially a single alkyl chain, may not sufficiently balance the multiheaded hydrophilic group, leading to the formation of a structure with behavior similar to typical hydrophiles); (ii) the mutual location of particular hydrophobic and hydrophilic groups—typically hydrophilic headgroups are gathered at the one side of the hydrophobic ones—but bolaform-type structures (i.e., hydrophilic groups that are symmetrically situated at both sides of the hydrophobic chain) and gradient surfactants (i.e., structures of amphiphilic character with gradually changing character from more hydrophilic at one end to more hydrophobic at the opposite one) may also be designed and synthesized; (iii) the choice of the initial block and terminal groups providing appropriate increments to the overall hydrophobicity and hydrophilicity of the structure as well as its symmetry and solubility in suitable solvents; (iv) and the utilization of proper reactions and their sequence, considering the durability of the formed linking groups (typically, labile ones may not be resistant to harsh conditions), the solubility of the particular intermediates in solvents, and the possibility of performing the intermediate purification/isolation steps. It should be emphasized that the choice of optimal alkyl chain length and structure constitutes one of the most important points for the design of surfactants characterized by the intended features. Such an issue may be exemplified by the complex research carried out by Verma et al. on corrosion protection surfactants. The findings include not only the simple influence of the length of the saturated hydrocarbon chain but also considerations on the packing of surfactant molecules on the surfactants (the impact of unsaturated hydrocarbons, planes of symmetry, aromatic rings, etc.) [29,30,31,32].

The starting (“initial”) building blocks for a surfactant with a single hydrophobic chain comprise, generally, simple and commercially available organic compounds, such as fatty alcohols or amine, carboxylic acid, or alkyl halide with a long hydrocarbon motif [22,33,34]. Conversely, the synthesis of the starting blocks for gemini-type or oligomeric-type surfactants may involve multistep reactions, providing the formation of a multifunctional compound containing multiplied hydrophobic groupings [35]. In general, the synthesis of the abovementioned derivatives comprises multiplied reactions such as alkylation of diamines (e.g., the reaction of an alkyl halide with ethylenediamine), Michael addition or amine–cyanuric anhydride coupling [22,36]. Such an approach desires appropriate purifications of the obtained intermediates to obtain the required purity of the final product. This process may be exemplified by the synthesis of *N*,*N*′-bis-alkyl, *N*,*N*′-bis(3-aminopropyl)ethylenediamine methanesulfonate–gemini-type G_0_ derivative, constituting a convenient starting point for hyperbranched surfactants [37]. The first step comprised the preparation of long-alkyl-chain dinitrile diamine utilizing Michael’s addition of ethylenediamine to acrylonitrile (1:2, mol:mol; solvent: mixture of acetonitrile with methanol; 50–60 °C, 36 h), followed by alkylation with 1-bromododecane (1:2, mol:mol; solvent: acetonitrile; reagent: K_2_CO_3_ (anhydrous); 86 °C for 38 h, then 140–145 °C for 6 h). The obtained intermediate consisting of the symmetric secondary diamine with two dodecyl alkyl chains and two terminal nitrile groups was diluted with soda lye and extracted with chloroform, followed by the drying of the organic phase with anhydrous K_2_CO_3_. The dinitrile semi-product was crystallized at −20 °C from hexane to give a white solid (melting point 25–27 °C, yield: 89.5%). Reduction of the dinitrile semi-product was performed in 1.5% NaOH solution in methanol using Ni (Raney) as the catalyst and gaseous H_2_ (300–500 psi) as a reducing agent. The reaction mixture was filtered, basified and extracted with chloroform, followed by drying of the organic phase with anhydrous K_2_CO_3_ and solvent evaporation. This diamine gemini-type semi-product was converted to suitable methylsulfonate salt by heating with an excess of methanesulfonic acid in an acetonitrile/methanol mixture, followed by filtration and recrystallization from an ethanol/ethyl acetate mixture, yielding high-purity dimethylsulfonate gemini-type surfactants. In order to obtain a free amine (semi)product, the methylsulfonate salt may be dissolved in soda lye and extracted with chloroform, while the organic layer may be dried with anhydrous K_2_CO_3_ followed by organic solvent evaporation to dryness [37]. It should be noted that repeating the steps above may lead to the formation of appropriate generations (G_1_, G_2_,…) of dendronium-type gemini surfactants.

Having discussed detailed information concerning the synthesis of the starting building blocks and relatively simple structures (e.g., gemini or biscephalous-type ones), it may be helpful to present general remarks considering the synthesis of particular types (groups) of multiheaded type surfactants. For oligomeric surfactants, i.e., those possessing multiplied hydrophilic and hydrophobic groups gathered together by appropriate linkers, the most common strategy involves the synthesis of a shorter or longer linker’s precursor containing a relevant number of reactive moieties (typically amine-type), followed by its quaternization with an excess of long-alkyl-chain halides. Such an approach comprises the use of well-defined amines (tri-, tetra-, etc., amines) in order to provide a surfactant characterized by a uniform structure and known molecular weight [6,7,38,39,40,41,42,43,44]. The synthesis of bolaform-type surfactants is, generally, very similar to their gemini-type analogues, although it utilizes α,ω-derivatives of hydrophobic building blocks, typically hydrocarbon-based or oligomeric siloxane. In order to avoid the formation of unsymmetrical single-chain single-tail surfactants, the usage of selective reactions (e.g., utilizing phase-transfer catalysts) or an excessive headgroup precursor reagent is highly encouraged [28]. It should be noted that applying the aforementioned strategies enables the synthesis of even very complicated structures if dendronium-type precursors are used. Therefore, the design of any dendronium-type surfactant should consider the results of the preliminary studies upon the simplest derivatives denoted as G_0_/G_1_ compounds, including typical single-head–single-tail surfactants, dicephalic (also known as bicephalous)-type structures, and gemini-type ones [20,45,46,47,48,49]. The significance of the design, synthesis and performance studies is crucial for further development of dendronium-type structures since low-molecular-weight surfactants are typically much easier to obtain in a pure form and are desirable for the physicochemical analyses, especially surface tension isotherms determination.

### 2.1. Modular Synthesis Based on Step-by-Step Reactions with Suitable Building Blocks

Modular synthesis based on step-by-step reactions is an approach comprising a group of synthetic routes that involve the initiator (e.g., hydrophobic tail’s source) and suitable building reagents, as well as enable the reproduction of the previous end group in the multiplied manner. In general, modular synthesis constitutes repeatable reaction steps with possible modifications of the first (exchange of the initiator reactive moiety) and terminal (formation of the actual hydrophilic group) steps [22,34,35].

**Scheme 2:**
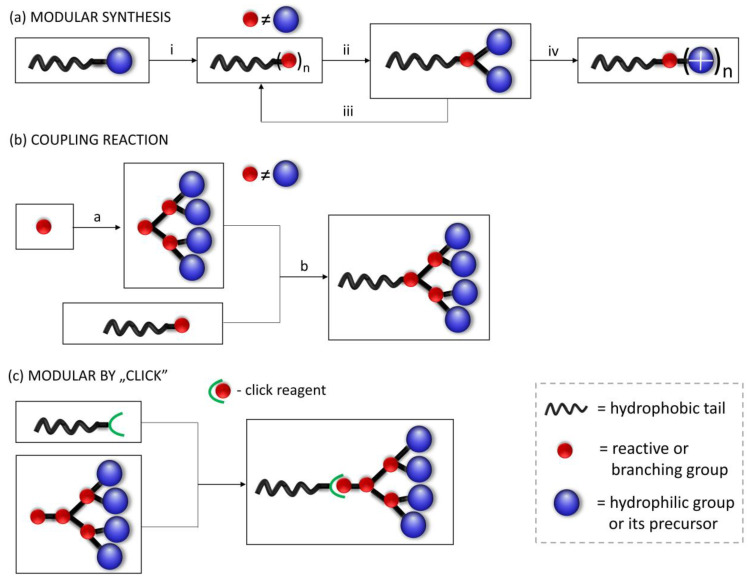
Synthetic approaches for multifunctional dendronium-type surfactants.

Modular synthesis (see Scheme 2a) may be divided into four steps: (i) the activation of (generally) the hydrophobic tail’s source or amphiphilic starting block; (ii) coupling with a symmetric reagent in order to introduce multiplied precursor groups for the branched structure/hydrophilic group; (iii) the exchange of the obtained precursors into reactive groupings; and (iv) the final modification of the terminal groups into multiplied hydrophilic motifs. Steps (ii) and (iii) are the most crucial, as they enable step-by-step reactions to form hyperbranched structures and precursors for hydrophilic moieties. It is worth noting that there should be a big difference between the reactivities of the intermediates after steps (ii) and (iii) to assume appropriate selectivity allowing for the formation of a dendronium-type hyperbranched structure. Such an approach leads to structure control by the number of repetitions of steps (ii) and (iii). The usage of typical bi- or multifunctional reagents (e.g., aziridine or allylamine) may ensure the preparation of hyperbranched or linear structures with poorly defined structures, e.g., containing both secondary and tertiary amine-linking moieties and different molecular weights [50,51]. It has to be highlighted that some requirements must be fulfilled by the chemical reactions utilized for (ii) and (iii) steps. In general, at least one building block should be multifunctional, possessing different moieties responsible for anchoring to the hydrophobic group or branch, as well as easy multiplication of novel branches/hydrophilic head groups. The reaction responsible for multiplication should be selective to assume the addition or substitution of the indented groups without damaging other ones. The second step—formation of the multiplied reactive groups for further modification—should enable gaining the particular number of reactive groups by proper hydrolysis, reduction, oxidation, etc. Moreover, steps (ii) and (iii) are preferably carried out under as mild conditions as possible, especially in terms of temperature. It is particularly important when geminal carboxylic acid groups are involved in the reaction, elevated temperature may lead to decarboxylation and the formation of only partially branched structures [52]. Furthermore, temperatures exceeding 180–200 °C may cause the chemical degradation of organic compounds coupled with the random formation of reactive double-bond motifs. To summarize, the design of the dendronium-type surfactants is particularly dependent on the considerations concerning step-by-step reactions as well as their conditions and initialization/termination steps.

Modular synthesis was exemplified (by the aforementioned carboxylate surfactants C18-G1-(COONa)_2_ and C18-G2-(COONa)_4_, synthesized and characterized by Lou et al. [22]) in Scheme 2, while the detailed information for the step-by-step approaches is provided in Table 1. This surfactant represents a typical procedure of a step-by-step approach involving a hydroxyl-terminated initial building block (octadecanol) reacted with 2,2,5-trimethyl-1,3-dioxane-5-carboxylic anhydride to form a dioxane-terminated derivative, hydrolyzed to the appropriate derivative with two terminal hydroxyl groups (step ii) [22]. In order to obtain a compound with terminal carboxylic acid salt groupings, the latter derivative was reacted with succinic anhydride, followed by acidic groups basification using sodium hydroxide in ethanol (step iii). It should be noted that the utilization of the reactivity of carboxylic acid anhydrides with hydroxyl groups (catalyzed by DMAP) provides high selectivity under mild conditions, while controlled hydrolysis (heterogeneously catalyzed) enables the easy duplication of terminal hydroxyl groups. The synthesis of amine-terminated dendrimeric-type surfactants (see example 2 in Table 1) may use fatty amine (i.e., hexadecylamine) as a starting building block [34]. The multiplied reproduction of terminal amine groupings is performed via repeated reactions with methyl acrylate (Michael addition under mild conditions; step ii) and ethylenediamine (amidation of ester moieties, step iii). It should be mentioned that the selectivity of both steps is assumed due to the suitable excess of reagents (threefold for Michael addition; twentyfold for amidation) as well as careful purification steps after each reaction (evaporation of solvents and low-molecular-weight reagent residues). The procedures are needed to provide high-purity products due to the possible reactions between traces of unremoved reagents (methyl acrylate and ethylenediamine). The synthesis of gradient-type dendrimeric surfactants may involve multistep reactions, utilizing cyanuric chloride and *tert*-octylamine as initial step building blocks (step i, see example 3 in Table 1) [36]. The latter derivative is reacted with 1,4-butanediamine and diisopropylethylamine to obtain an intermediate derivative (step ii) for further modification with cyanuric chloride and diisopropylethylamine (step iii). The final treatment (step iv) includes modification with Jeffamine EDR-192 and diisopropylethylamine to tune the hydrophilicity/hydrophobicity of the final derivative. It is noteworthy that the procedures described in examples 2 and 3 in Table 1 may be easily tuned for the synthesis of gemini-type dendronic surfactants (see examples 4 and 5 in Table 1) [34,36]. In general, the main difference is the dichain character of the starting building block and the molar ratio of reagents. The reaction conditions are very similar to their “mono” analogues, while the purification steps are the same. Such findings show the high usefulness of step-by-step reactions. In addition, usually, Michael’s addition reactions (see examples 2 and 5) are performed using a low-molecular-weight solvent (methanol) at an only slightly elevated temperature (40 °C) for a long time (50–90 h). Amidation reactions (see examples 2 and 5) are also performed in methanol at 40 °C, but the time needed for their completion is longer (80–140 h). Such conditions are profitable for highly selective reactions due to the limited risk of degradation of high-molecular-weight reagents. Reactions involving 1,4-butanediamine and diisopropylethylamine (see examples 3 and 4) are performed at elevated temperatures (40 °C) in THF while coupling with cyanuric chloride and diisopropylethylamine needs initial cooling to 0 °C. To summarize, all synthetic routes for the step-by-step synthesis of dendronium-type surfactants involve reactions carried out under mild conditions (temperatures not exceeding 100 °C) using a volatile organic solvent that enables easy purification steps due to evaporation under reduced pressure. Such strategies provide the formation of high-purity products.

### 2.2. Coupling of Hydrophilic Surfactant Group with Hydrophobic Chain Utilizing the Selective by-Product Free Reaction

In contrast to modular synthesis, it is possible to separately synthesize hydrophilic (or amphiphilic) dendronium-type derivatives with an adequate reactive group and source of hydrophobic moiety (typically: an alkyl chain) [33,45,53,54]. The latter derivatives are subsequently coupled to form appropriate surfactants with dendronic headgroups. The latter derivatives are subsequently coupled to form appropriate surfactants with dendronic headgroups. See Scheme 2b for the schematical representation (step a—synthesis of the dendronium-type hydrophilic group, step b—coupling the hydrophilic fragment with hydrophobic chain).

The reaction utilized to couple hydrophilic (or amphiphilic) dendronium-type fragments with a hydrophobic derivative should fulfill several criteria: (i) sufficient selectivity to provide the formation of one needed chemical bond without any modification of other groupings in both fragments; (ii) the formation of an exclusively desired amphiphilic product without any by-products—even low-molecular-weight stoichiometric by-products may be adsorbed/absorbed within dendronic surfactants, leading to obtaining a poor-quality product; (iii) any additional substances (solvents, catalysts and initiators) should be easily removed from the reaction mixture; (iv) the overall yield should be close to 100% in order to avoid the difficult removal of unreacted substrates. Feature (i) is the most crucial, as it allows for the creation of high-quality products—a dendronium-type surfactant instead of an amphiphiles mixture. Such approaches are highly desired for any fine chemical products since they enable the selective formation of the designed amphiphile with high yield and purity without using multistep purification processes of limited efficacy toward amphiphiles.

In general, one of the most common approaches comprises the protection of the appropriate chemical group followed by coupling under deprotecting conditions. Typically, a dendronium-type substrate is characterized by the presence of multiplied chemical bonds, especially labile ones (e.g., amide or cyclic acetal). Hydroxyl groups, especially geminal ones, need to be protected by the transformation to acetal (see example 1 in Table 2) [45] or methylation (see example 3 in Table 2) [54,55]. Deprotection conditions may involve heating with aqueous trifluoroacetic acid (see example 1 in Table 2) or a reaction with the same reagent performed in polar organic solvent (methanol) at room temperature (see example 1 in Table 2). Such a reaction provides the removal of protective acetal (or ketal) groups, including their cyclic derivatives and the formation of geminal diols for further reaction or as terminal groupings of hydrophilic character. On the other hand, the mesylation of phenolic moieties allows for suitable protection and sufficient reactivity under basic conditions (K_2_CO_3_ in anhydrous dimethylformamide) to enable the generation of an ether bond (aromatic–aliphatic ether)—see example 3 in Table 2 for further information. It should be noted that the abovementioned process comprises a single-step reaction completed via refluxing the reagents under a protective, inert atmosphere for 24 h. However, protection and deprotection lead to the preparation of (generally) low-molecular-weight side products. In order to assume reaction completion, it may be necessary to utilize a high excess of one reagent (see example 3 in Table 2). Moreover, the complexity of the whole process can lead to poor total yields (typically below 50%). Those issues constitute the reason for multistep purification processes involving the evaporation of organic solvents/reagents under reduced pressure, followed by gel chromatography.

A significant drawback of typical organic procedures for coupling the hydrophilic, dendronium-type motif with hydrophobic moiety is in the significant demand for very selective and irreversible single-step reactions [33,53]. Such novel approaches may be performed using appropriate “click” chemistry reactions or Michael’s addition. It has to be mentioned that Michael’s addition may be performed without any catalyst and even under solvent-free conditions (e.g., for acrylate esters) in comparison to “click” reactions. Such a procedure can significantly enhance the yield of the overall process and limit purification to the removal of the solvent and/or excess of the reagent under reduced pressure. These novel approaches, comprising very promising strategies for the synthesis of multiheaded surfactants, are described in detail in Section 2.3. 

**Table 2:** Examples of coupling of hydrophilic surfactant group with hydrophobic chain utilizing selective by-product free reaction.

No.	Dendronium Type Hydrophilic/Amphiphilic Group	Hydrophobic Moiety	Coupling Conditions	Refs.
1	Glycopolymer	Polyvinylamine	(1) Deprotection of glycopolymer via reaction with 1% trifluoroacetic acid at 55 °C for 1 h (2) Reductive amination using sodium cyanoborohydride	[45]
2	Appropriate Boc-protected (amine terminal groups) dendronium-type glicydyl ester—generations G0–G3	1-prop-2-ynyloxy-octadecane	(1) Azide-yne “click” reaction utilizing DIPEA and bromotris (triphenylphosphine)-copper(I) as catalysts in THF (40 °C for 1 h); reaction control—TLC; purification—column chromatography (CHCl_3_/MeOH, 99:1) (2) Deprotection of terminal amine groups and their protonation with trifluoroacetic acid	[33,53]
3	Third-generation glycerol dendron protected with mesyl groups	(E)-4-((4-(undec-10-enyloxy)phenyl)diazenyl)phenol	(1) O-alkylation of hydroxyl group in phenol moiety; solvent: dry DMF; reagent: anhydrous K_2_CO_3_; 3-fold excess of dendron; room temperature for 45 min (before dendrion was introduced); 130 °C for 24 h under argon; solvent evaporation followed by extraction from CH_2_Cl_2_/H_2_O system; purification–column chromatography (hexane/ethyl acetate, 4:1; silica gel) (2) Reagent–trifluoroacetic acid (high molar excess); solvent: methanol; room temperature, 5 h; solvent evaporation under reduced pressure	[54,55]

DIPEA: *N*,*N*-diisopropylethylamine; THF: tetrahydrofuran; DMF: dimethylformamide.

### 2.3. Coupling via Click Synthesis

Chemical reactions for coupling hydrophilic surfactants’ groups with hydrophobic chains may involve numerous processes, such as reductive amination utilizing sodium cyanoborohydride [45], “click” reactions [33,53] as well as the formation of an azo bond between two aromatic rings [54,55]. The usefulness of “click” reactions should be described more carefully. In general, “click” reactions apply to very selective reagents providing the formation of stable, covalent bonds without any by-products [56,57,58,59]. Two “click” reactions have gained significance in organic synthesis: thiol–ene [60,61,62] and azide-yne ones [57,58,59]. Stable S–C bond generation via the reaction of a thiol group and a double bond comprises a well-known reaction, but it became very common when free-radical conditions were examined for its profound selectivity [63,64,65]. On the other hand, the formation of a strong triazine ring by the reaction of azide with a triple bond was first named a “click” reaction in the early 2000s [58,59]. This process is catalyzed by Cu(I) ions, enabling reaction completion without the presence of by-products. It should be noted that reactions between obsolete chemical groups, rarely present in typical organic compounds, provide the highest selectivity due to the inability to attract any other existing groups, such as amine, hydroxyl, halide, or carboxylate [57,58,59]. Such an approach opens new possibilities to apply very selective reactions in organic synthesis, ensuring direct and clean (i.e., without by-products) coupling of two reagents, thus avoiding unnecessary steps, including protection and deprotection. Such an approach opens new possibilities to apply very selective reactions in organic synthesis, ensuring direct and clean (i.e., without by-products) coupling of two reagents, thus avoiding unnecessary steps, including protection and deprotection. See schematical representation in Scheme 2c.

The usefulness of azide-yne “click” reactions may be exemplified by the coupling of amine-terminated, Boc-protected, dendronium-type glycidyl esters with 1-prop-2-ynyloxy-octadecane [33,53]. The reaction catalyzed using bromotris (triphenylphosphine)-copper(I) and *N*,*N*-diisopropylethylamine was completed within 1 h under mild conditions (tetrahydrofuran as the solvent, 40 °C; monitored by TLC). A purification step, including column chromatography using a mixture of chloroform with methanol (99:1) as a mobile phase, was needed to remove the catalyst. Despite the very selective character of azide-yne “click” reactions, terminal amine groups in the dendronium-type headgroup are protected by Boc, so the last step involves their deprotection and protonation using trifluoroacetic acid. It is worth noting that the usage of thiol-ene “click” reactions, although very common in surface modification and polymerization, is limited for surfactants due to the free radical mechanism involving multiple steps that may lead to internal isomerization, followed by the formation of different product mixtures.

The similarity between “click” reactions and Michael’s additions, especially in the field of mild reaction conditions, profitable kinetics and a lack of any by-products, enables us to discuss both processes together [28,60,66]. Typically, Michael’s addition (especially the reaction between acrylates and amine bonds) is significantly useful for modular synthesis (see details in Section 2.1), although it is possible to use it for coupling reactions, especially when a hydrophile-bearing amine is attached to long-alkyl-chain acrylate. Such an approach is particularly applicable to the synthesis of single-head double-tail derivatives of methylamine—the final step involves only the quaternization of the obtained tertiary amine [67]. The application of Michael’s addition for the synthesis of dendronium-type surfactants via coupling reactions comprises one of the most challenging and useful strategies, especially due to the mild reaction conditions, high selectivity and lack of any by-products.

## 3. Self-Assembly of Multicharge Cationic Surfactants

### 3.1. Nonequivalent Adsorption at the Air/Solution Interface 

**Figure 1:**
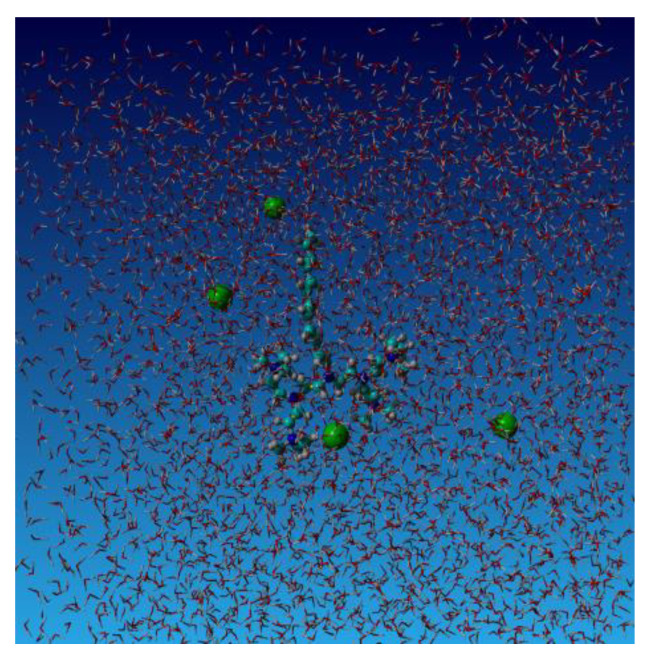
Snapshot from the molecular dynamics simulations of *N*,*N*,*N*-bis(2-(*N*,*N*,*N*-bis(2-ammoniumethyl)amino)ethyl)dodecylamine tetrabromide surfactant in water. Green spheres illustrate bromide counterions.

In general, ionic surfactants that gather at the interface cause its charging and the formation of an electrical double layer (EDL). The surface charge of the adsorbed surfactant induces electrostatic repulsion with the molecules remaining in bulk, influencing the free energy of their further adsorption. In general, multicharged surfactants are less surface active than their monovalent counterparts, as was demonstrated for dicephalic surfactants [13]. The effect specific to multicharged surfactants is the formation of transient complexes with their counterions (or ions of the opposite sign of the added salt). This effect corresponds to the well-known counterion condensation for polyelectrolytes [68] and affects the adsorption at liquid interfaces since their effective charge can be lower than a nominal one. That was demonstrated for divalent cationic surfactants, the bis-ammonium salts, bis[2-hydroxy-3-(dodecyldimethylammonio)propyl]-alkylamine dichloride and trivalent ones, tris-ammonium salts, bis[2-hydroxy-3-dodecyldimethylammonio)propyl]-dialkylammmonium trichloride [69]. The determined surface tension isotherms indicated the lack of significant differences in surface activity between those bis- and tris-ammonium salts, contrary to the expectations for divalent and trivalent surfactant ions. That effect was explained by assuming the formation of multiple surfactant ion–counterion associates, which was evidenced by the results of the measurements of the concentration of free chloride anions in the surfactant solution. The formation of transient multicharge surfactant/counterion complexes can also be evidenced by the dynamic molecular simulations [13], as illustrated in Figure 1, which demonstrates the snapshot of simulations for *N*,*N*,*N*-bis(2-(*N*,*N*,*N*-bis(2-ammoniumethyl)amino)ethyl) dodecylamine tetrabromide, a surfactant having four quaternary ammonium groups, therefore, bearing positive charge +4. The size of the simulation box corresponded to the surfactant concentration of 3 × 10^−10^ mol/dm^3^. The results of the simulations indicate that for 50% simulation time, the surfactant is complexed by one bromide counterion and for 2% by two of them. The YASARA Structure simulation package was used with the AMBER14 force field [70].

The general problem of the model approach of ionic surfactant adsorption is the description of the electrical interactions between adsorbing charged species. We have recently presented a comprehensive review of existing approaches for describing adsorption at liquid interfaces that could be useful for multiheaded cationic surfactants of various architectures—structures possessing multiple polar headgroups (e.g., dicephalic, dimeric (so-called gemini), trimeric, tetrameric, etc.) [1]. In particular, the previously developed Surface Quasi-Two-Dimensional Electrolyte (STDE) model of surfactants adsorption at the water/air interface [71] and later expended [1,11,72] is convenient for describing the adsorption properties of a variety of multiheaded cationic surfactants. Recalling, the STDE model assumes that, due to the strong electric field, the counterions can penetrate the Stern layer, treated as a surface quasi-two-dimensional electrolyte (STDE), in which the electroneutrality condition is not met. For multicharged surfactants, Stern layer penetration is particularly pronounced. A detailed description of the STDE model and its application for monovalent ions are presented in the above-cited reports by Warszyński et al. Further discussion of the application of the model for the description of the adsorption of multicharged surfactants can be found in our review [1].

**Figure 2:**
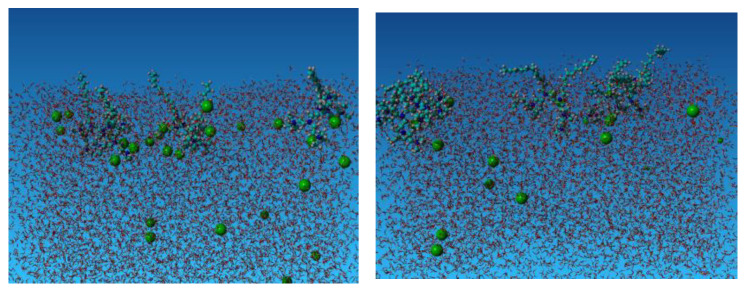
Snapshots from the simulation of adsorption of *N*,*N*,*N*-bis(2-(*N*,*N*,*N*-bis(2-ammoniumethyl)amino)ethyl)dodecylamine tetrabromide (**left**) at the water–air interface illustrating the presence of bromide counterions at the surface, in agreement with the assumption of the STDE model of ionic surfactants adsorption (**right**). Simulations were performed with the 32bit version of YASARA Structure software package.

Molecular dynamics simulations of multicharge surfactants at the air–water interface have also been performed to obtain a better understanding of the self-assembly processes of multicharge surfactants and have provided support to the STDE approach [73]. In particular, molecular dynamic simulation allows for substantiating the main assumption of the model, the penetration of the Stern layer by the counterions. That is illustrated in Figure 2, which shows snapshots from the molecular simulation run of the adsorption of *N*,*N*,*N*-bis(2-(*N*,*N*,*N*-bis(2-ammoniumethyl)amino)ethyl) dodecylamine tetrabromide and its gemini counterpart *N*,*N*-ethane-1,2-diylbis(*N*-dodecylethane-1,2-bis(*N*-(2-*N*,*N*,*N*-trimethylammoniumoethyl)ethane-1,2-diamine)-diamine) tetrabromide.

### 3.2. Aggregation in Aqueous Solution

The self-assembly of a variety of multifunctional ionic surfactants in aqueous solution is essential to their application and is characterized by aggregation ability and aggregate morphology. They show strong self-assembly forming various aggregate structures ranging from the nano- to the microscale and reveal desirable interfacial activity and miscellaneous phase behavior [20]. It is generally known that in the bulk aqueous phase, surfactants above their critical micellar concentration (CMC) spontaneously form aggregates, such as micelles, where hydrophobic tails are located in the micellar inner core, and the hydrophilic head groups are in contact with the aqueous phase [74]. Depending on the surfactant architecture, the shape and size of such aggregates can thus vary significantly from micelles to spheroidal micelles to vesicles to hexagonal aggregates, etc. [4,6,75]. The presence of multicharge head groups could enhance the strength of the interaction between the cationic moiety and the water molecules, increasing their solubility in water and making them strongly interact with counter anions. Therefore, along with the increasing number of head groups, the ratio of hydrophilic to hydrophobic interaction strength increases, and as a consequence, the aggregation number of surfactants in the aqueous solution noticeably decreases, leading to smaller micelle formation. Furthermore, a variety of polydisperse aggregates may appear in the aqueous environment [38,40,43,44].

Generally, ionic surfactant structures consist of hydrophobic and hydrophilic parts. Depending on many controlled parameters such as the multicharge surfactant structure, the hydrophilic or/and hydrophobic entities number, the type and chemical reactivity of the linker connecting the hydrophobic and hydrophilic parts, the designed functional surfactant architecture, the assumed concentration of surfactant ions, counterions and other additives, the features of chemical environment and the temperature magnitude, they self-assemble into a broad range of morphologies [75,76]. Such aggregation processes are governed by their attractive hydrophobic interactions between the hydrophobic segments and repulsive steric or electrostatic interactions between the hydrophilic head groups [77]. Israelachvili et al. elaborated a model-based approach that can foresee the kind of aggregate morphology depending on the geometrical constraints of the individual aggregate building components [78,79]. Accordingly, the packing parameter for the studied surfactants can be calculated in order to elucidate the morphology of the aggregate formed at the interfaces and in the solution. It must be emphasized that the packing parameter, although very suitable to rationalize self-assembly from a qualitative point of view, provides quantitative guidance for experiments. In order to predict more quantitative measures, it is advisable to apply more explicit molecular-level theory [80,81], computer simulations [42,43,82] or numerical self-consistent field models [21,83]. In particular, the molecular dynamics simulations seem promising in predicting the aggregation properties of multicharged surfactants. For example, both the coarse-grained simulation of the aggregation properties of multiheaded cationic surfactants in water [42] and the all-atom simulations indicate that the aggregation number and the size of micelles decreased with the number of charges in the hydrophilic headgroup [43]. That is in agreement with the experimental findings of Haldar et al., who used small-angle neutron scattering (SANS) to investigate the aggregation number of surfactants having a single hydrocarbon tail (C_14_) with one, two, or three cationic quaternary amine groups with ester linkers [38] and can be explained by the increased electrostatic repulsion between headgroups. However, systematic research on the effect of counterion condensation (effective micelle charge) and counterion specificity [13] should be further performed.

The interesting species are also tadpole-type amphiphilic dendrimers, comprising hydrophobic chains and hydrophilic dendrons, forming aggregates of different solution shapes. Yoshimura et al. synthesized dendrimers featuring a hexadecyl chain and poly(amidoamine) dendrons and thoroughly investigated their surface activity and association behavior [34]. These amphiphilic dendrimers can create a new category of surfactants with multiple polar heads exhibiting unique aggregation behavior.

### 3.3. Self-Assembly with Oppositely Charged Species

One of the most effective methods for the further modification of adsorption or aggregation properties of multihead surfactants properties that open the possibility of numerous applications is the insertion of oppositely charged additives, including other surfactants, organic or inorganic salts and polymers [20]. Such compounds influence the hydrophobic interactions between the alkyl chains and the electrostatic interactions among the charged head groups of surfactants. It is well known that the additives can strongly affect the self-assembly behavior of the surfactants in aqueous solution and their molecular conformation.

Oppositely charged compounds can impair the repulsive forces between the polar heads of the surfactant, strengthening the self-assembling structures, which are more stable than in a solution of free surfactants [6,40]. The addition of inorganic salts leads to the screening of the electric charge of surfactant headgroups and decreases the electrostatic interaction between them. As described above, the screening is ion-specific. The reduction in electrostatic repulsion induces changes in the size and geometry of aggregates. The presence of counterions stabilizes the self-assembling structures of the ionic surfactants, promoting their growth and the molecular conformation transition, inducing the formation of aggregates with various non-spherical shapes from stretched to pyramid-like.

Two mixed surfactants of the same charge promote micelle formation; however, the surfactant mixed with oppositely charged species leads to vesicle, lamellae or higher ordered aggregate structures formation. Mixtures of surfactants of opposite charge, referred to as catanionic surfactants, exhibit cooperative adsorption and aggregation behavior due to the electrostatic attraction of their headgroups [84]. Therefore, they may exhibit extremely low CMC, one or two orders of magnitude lower than single ionic surfactants with equivalent concentrations. These self-assembled surfactant structures contain rich hydrophobic domains, making their application very promising in various industries, such as oil recovery [85,86] or pharmacy [87]. However, the problems of easy precipitation and phase separation in most catanionic systems have greatly restricted their practical applications [88].

The self-assembly of charged surfactants with oppositely charged polyelectrolytes offers another possibility to form amphiphilic structures with a variety of possible applications [52,54,55,89,90,91,92,93]. Upon complexation of polyelectrolytes with the oppositely charged surfactants, they acquire surface activity and absorb liquid/gas, manifesting as surface tension decreases.

Penfold and Thomas proposed the macroscopic model of the surface tension of ionic surfactant/polyelectrolyte mixtures [91]. They recognized two types of adsorption behavior that depend on the strength of surfactant polyelectrolyte interactions. The first one is observed for systems where the interactions of surfactant and polyelectrolyte are strong, and the surface-active polyelectrolyte/surfactant complexes adsorb strongly at the air–water interface and can form thick layers. With the increase in surfactant concentration, the surface tension of the mixture decreases until it reaches a constant value at a surfactant concentration named CAC (critical aggregation concentration). The further addition of surfactant produces only a slight decrease in the surface tension until the surfactant concentration equal to its CMC (critical micelle concentration) is reached. In that range of concentrations, polyelectrolyte-mediated formations of surfactant aggregates occur. If the interaction of the surfactant and polyelectrolyte is weaker, another type of adsorption behavior is observed. At a certain surfactant concentration between CAC and CMC, a sharp peak in the surface tension appears. Then, the surface tension approximately follows the surface tension isotherm of pure surfactant when its concentration is increased.

Since the interactions of multicharge surfactants with the oppositely charged polyelectrolytes are strong, the first type of adsorption behavior prevails. That was demonstrated for the cationic gemini surfactant N-dodecyl-3,3′-imino-bis(*N*,*N*-dimetylopropylammonium) bromide or dicephalic-type surfactant *N*,*N*-bis[3,3′-(trimethylammonium)propyl]-dodecanamide di-methylsulfate and anionic polyelectrolyte polystyrene sulfonate (PSS) [94]. Similar behavior at the water/oil interface was also observed [94]. The observed CAC is more than two orders of magnitude lower than CMC. Therefore, in the presence of polyelectrolytes, the formation of micellar aggregates able to solubilize hydrophobic cargo requires a much lower surfactant concentration. That makes polyelectrolyte–surfactant complexes (PESCs) promising systems for drug delivery.

#### Polyelectrolyte–Surfactant Complexes as a Delivery Platform for Poorly Soluble Drugs

As described above, ionic surfactants can interact favorably with oppositely charged polyelectrolytes either through strong electrostatic, hydrophobic or intermolecular interactions involving polar groups, and such systems have frequently been studied by experiments and simulations [89,90,91]. Accordingly, the interaction force between the surfactant and electrolyte in PESCs depends on the choice of both compounds (PE and surfactant), the average molecular mass of PEs, the nature of polar groups, pH value, charge, length of the surfactant alkyl chain, nature of the hydrophilic group and other factors. The abovementioned interactions enable a strong binding of the surfactant molecules or surfactant aggregates to the polyelectrolyte, leading to the formation of a rich diversity of network structures of soluble PESCs that depend on several PE and surfactant parameters. The crucial factors with respect to PE are the chain stiffness, the charge density along the backbone and the molecular weight. The most important surfactant features are the type of head group and hydrophobic part, as well as the packing parameter, which indicates the type of preferentially formed aggregate. Apart from that, the mixing ratio of PE and surfactant, total concentration and external factors, such as temperature, pH, pressure, ionic strength and added cosolvent, have an influence on the PESC structure [89,95]. Depending on all these parameters, various nanostructured complexes can be achieved, from spherical micelles bound by PE to ordered multi-layered structures. The PESC self-organized aggregates are very versatile, and the presence of surfactant systems makes them possible to solubilize hydrophobic compounds due to the formation of small hydrophobic domains of aggregates (interior of aggregates). Combining the solubilization properties of surfactants of a small size (3–8 nm) with polymers of a larger size (from 20 nm to several μm), PESC structures ranging from 10–20 nm to many thousands nm can be formed depending on the PE length and the number of micellar aggregates [96]. Interactions between polyelectrolytes (PEs) and oppositely charged ionic surfactants, and the fabrication of PESCs, have been thoroughly investigated in recent decades due to the particular interest in nanostructured self-assembled systems and their unique physical mechanisms of formation [89,95,96,97,98,99]. Polyelectrolyte–surfactant complexes have many industrial and technological applications in the field of food, pharmacy, cosmetics, paints and detergents. The widespread usage of PESCs is associated with their specific properties and behavior at different interfaces that surfactants alone cannot provide. PESCs attract much attention in pharmaceutical research regarding their ease of formation, appropriate biocompatibility and satisfactory drug loading [99]. The application of PESCs gives an opportunity to generate formulations with high flexibility in relation to their structure and rheological and solubilization properties.

The formation of polyelectrolyte–surfactant complexes allows for the construction of various aggregates with slower response times than pure surfactant systems which may possess good solubilization features but release active substances too fast. Thus, PESCs are suitable for drug delivery as they are able to control the release rate and modulate the release profiles of enclosed biologically active compounds. Moreover, the preparation of formulations with appropriate properties requires the usage of compatible surfactants and PE with minimal toxicity. In that context, the application of PESCs formed above the critical aggregation concentration which is lower than the critical micelle concentration reduces the corresponding toxicity. Thus, the presence of polyelectrolyte not only stabilizes the PESCs and improves solubilization but also minimizes the free surfactant concentration, which is essential for planned drug delivery applications [96]. Another advantage of PESCs and the interactions between them concerning drug solubilization is the ability to generate various solubilization sites, as hydrophobicity and polarity within the complex occur at different places. The solubilization of drugs within the PESCs may result from hydrophobic or electrostatic interactions [90,99]. The region between the headgroup of surfactant and polyelectrolyte charge (interionic complex) is an attractive site to solubilize more polar drug molecules. The choice of the surfactant’s hydrophobic chain and the charge groups of the surfactant and polyelectrolyte allows for tuning drug solubilization sites. Multicharge surfactants that strongly interact with polyelectrolyte chains may form stable hydrophobic domains, which could be the most profitable for solubilization (see Table 3). Additionally, the complexes of these surfactants with Pes ensure the faster solubilization of hydrophobic substances compared to pure micellar systems.

Although many studies describing the interaction of conventional single-chain surfactants with polyelectrolytes have been reported, the multicharged surfactants proved to possess superior properties to form complexes with polyelectrolytes [100,101]. It is worth noting that gemini and dicephalic-type surfactants comprise G_0_ and G_1_ generations for dendronium-type structures of geminal or single-tail single-head architectures; therefore, they may be considered the essential structures for the above-mentioned investigations. On the other hand, there is also a limited number of studies concerning the interactions between oligomeric surfactants and amphiphilic polymers [69]. The multicharged surfactants have unique properties, including good solubilization, low Krafft temperatures, low critical micelle concentration (cmc), great rheological behavior, as well as high efficacy in lowering the surface tension [101,102,103,104,105,106,107,108,109]. Gemini surfactants were the most commonly complexed with a DNA molecule to form efficient carriers for gene therapy. For instance, D.R. Acosta-Martínez et al. studied the DNA complexation with a series of bis-quaternary ammonium gemini surfactants with varying alkyl chain spacers of 4, 6 and 14 carbons (GS4, GS6 and GS14) [106]. They demonstrated the effect of surfactant hydrophobicity on the DNA complexation process, cytocompatibility and DNA transfection, thus developing effective gene carriers. The related work describing the influence of spacer length and the presence of a polar head group of gemini surfactant on the complexation process was one for the systems of dsDNA and 3,3′-[α,ω-(dioxaalkane)]bis(1-dodecylimidazolium)chlorides [107]. It was observed that the studied complexes formed a variety of spatial structures, such as micellar, hexagonal and cubic. The cytotoxicity study has revealed that the surfactants used are safe for the HeLa cell, confirming that the formed PESCs can be applied as efficient DNA carriers. An interesting example is the formation of a complex composed of alkanediyl-α,ω-bis[(oxymethyl)dimethyldodecylammonium] chlorides and nucleic acid oligomers, such as dsDNA and siRNA [108]. It was found that the studied microstructure complexes had predominantly micellar or cubic forms, and the complexation process was more efficient toward siRNA. The performed cytotoxicity tests on HeLa cells showed that the dicationic gemini surfactants formed with siRNA and dsDNA stable systems could be applied as carriers for the transfection of therapeutic nucleic acids.

In the context of the development of natural formulations, the use of biopolyelectrolytes in the complexation with surfactants has become an interesting approach due to their non-toxic effect, biocompatibility, biodegradability as well as specific molecular architecture [95]. Lately, M.A. Bhat et al. published an article in which they used carboxymethylcellulose, succinic acid, as a biocompatible cross-linker, and gemini surfactant—ethane-1,2-diyl-bis(*N*,*N*-dimethyl-N-dodecylammoniumacetoxy)—to obtain a hydrogel system for quercetin encapsulation [110]. The investigation demonstrated noticeable modulation in viscoelastic, mechanical and self-healing properties. The prepared hydrogels showed the ability to release the drug in a sustained and controlled manner. The biocompatibility of the studied surfactant makes the hydrogel systems suitable candidates for drug delivery. Another study addressed the case of using methylcellulose and alkanediyl-α,ω-bis(dimethylcetylammonium bromide) surfactant for the complexation of rifampicin as the model hydrophobic drug [111]. In this work, the role of the surfactant spacer length on the physicochemical properties of PESCs, such as viscosity, turbidity, gelation temperature and solubilization, was examined. The study revealed that the gemini surfactants strongly affect the interactions between polyelectrolyte and the drug rifampicin. It was proven that the functional features of PESCs can be controlled by manipulating the structure of the surfactants. The importance of polyelectrolyte–surfactant system formation was also showed for *N*,*N*-bis[3,3′-(trimethylammonio) propyl] dodecanamide dimethylsulfate complexes with carrageenan, dextran and poly(sodium 4-styrenesulfonate) that are able to form nanocapsules encapsulated with hydrophobic cyanine-type photosensitizers, IR-786 and IR-780, model hydrophobic dye, Oil Red O, as well as the amphiphilic drug daunorubicin [94,112,113]. In these works, the impact of dicephalic surfactant polyelectrolyte interactions on the stability and permeability of nanocarriers was investigated. The studied systems proved to form efficient nanocapsules with the sustained and long-term release of active molecules.

An interesting feature of PESC formulations for drug delivery is the control of their rheological properties. For some drug delivery systems, enhanced viscoelasticity is needed. Normally, PESC aggregates exhibit viscosity close to that of water, but it can be altered when the interconnection of the polyelectrolyte–surfactant complexes occurs, leading to the structural variety of PESCs due to electrostatic interactions, hydrophobic interactions or H-bonding. Using such interactions, it is possible to form systems with improved viscosity properties. For example, the addition of hydrophobically modified polyacrylamide to the cationic gemini surfactants (2-*N*,*N*′-bis(dimethyloctadecyl) ethene ammonium bromide) enhanced the viscoelasticity via the formation of self-assembly networks of wormlike micelles [114]. It should be noted that the increase in viscosity to a gel-like system can be obtained by using a chemically cross-linked polyelectrolyte gel. In this situation, a surfactant is added to the initially present polyelectrolyte, forming a network structure whose rheological properties are controlled by PE gel. In this context, some polyelectrolytes, such as hyaluronic acid or hyaluronan, initiate highly viscous aqueous solutions at low concentrations due to the occurrence of physical cross-linking. The rheological features of PESCs systems can also be easily tuned by external physical or chemical stimuli, including pH, temperature, pressure, ionic strength, light, magnetic or electric field, etc. The viscosity response of such complexes allows them to create tailor-made pharmaceutical formulations, for instance, drug delivery systems whose viscoelastic properties might be modified upon pH or ionic strength changes. An interesting case of rheologically responsive PESCs is a combination of ethyl(hydroxyethyl) cellulose and two gemini arginine-based surfactants (*N*,*N*-bis(N-acylarginine)α,ω-dialkyl amide) to produce a thermoresponsive hydrogel with low toxicity. The viscosity of such a system was dependent on the temperature as well as surfactant concentration. The study revealed that a much lower concentration of cationic gemini surfactants compared to single-chain surfactants was required to form gel systems with appropriate biocompatibility [115]. Another advantage of PESCs with respect to drug delivery systems is their ability to construct drug-encapsulating formulations whose stability depends on pH. Via those means, the change in pH leads to aggregate disintegration and cargo release. An interesting research area is the application of stimuli-responsive polyelectrolyte–surfactant complexes as smart foams in the cosmetic and pharmaceutical industries. A good example of such development is the mixture of polyacrylic acid and cationic surfactant gemini 12-2-12 that forms very stable foams with controlled pH responsiveness [116].

Among the multihead surfactants, the oligomeric ones were mainly described in accordance with their self-assembly with polymers, in particular hydrophobically modified (HM) polymers. Mixing oligomeric surfactants with HM polymers improves the aggregation ability, induces the formation of structures with richer aggregate morphology and enhances the solution and interfacial properties. Until now, the effects of multicharged surfactants of various shapes on their mixtures with HM polymers were studied and thoroughly described in the article prepared by Fan and Wang [20]. It was found that the polymers influence the variation in the molecular conformation of multihead surfactants and provide unique properties of oligomeric surfactants, allowing for the development of multifunctional surfactants with advanced applications.

**Table 3:** Exemplary polyelectrolyte–surfactant complexes for drug delivery applications.

No.	Surfactant Type	Surfactant	Polyelectrolyte	Solubilized Drug	Studied Properties	Ref.
1	Gemini-type structure 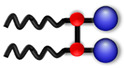	GS4–1,4-Bis(tetradecyl dimethyl ammonium)butane dibromide GS6–1,6-Bis(tetradecyl dimethyl ammonium)hexane dibromide GS14–1,14-Bis(tetradecyl dimethyl ammonium)tetradecane dibromide	DNA	-	Self-assembly properties; cytocompatibility (HeLa cells); DNA transfection; thermodynamic characterization	[106]
2		Alkanediyl-α,ω-bis [(oxymethyl)dimethyldodecylammonium]	dsDNA siRNA	-	Morphology, binding capacity, conformation, structural parameters; cytotoxicity (HeLa cells)	[108]
3		Ethane-1,2-diyl-bis(*N*,*N*-dimethyl -N-dodecylammoniumacetoxy)	Carboxymethyl -cellulose (CMC)	Quercetin	Viscoelastic and mechanical properties; self-healing properties; structural and morphological properties; thermal properties; drug release studies	[110]
4		Alkanediyl-α,ω-bis(dimethyl -cetylammonium bromide)	Methycellulose (MC)	Rifampicin	Surface tension; viscosity; turbidity; gelation temperature	[111]
5		Gemini 12-2-12	Polyacrylic acid (PAA)	-	Equilibrium and dynamic surface tension; surface dilational rheology; stability studies; pH responsiveness	[116]
6		(*N*,*N*-bis(N-acylarginine) -α,ω-dialkyl amide)	Ethyl(hydroxylethyl) cellulose (EHEC)	-	Thermoresponsiveness; rheological properties; cytotoxicity (HeLa cells)	[115]
7	Dicephalic-type structure 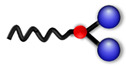	*N*,*N*-bis[3,3′-(trimethylammonio)propyl] dodecanamide dimethylsulphate	Carrageenan (CAR)	Photosensitizer–IR-786	Interfacial tension; stability; permeability; drug release studies	[112]
8		*N*,*N*-bis[3,3′-(trimethylammonio)propyl] dodecanamide dimethylsulphate	Dextran (DEX)	Daunorubicin	Morphology; stability; drug release profiles; cellular internalization; cytotoxicity (colon carcinoma cells); proapoptotic and hemolytic activity	[113]
9		*N*,*N*-bis[3,3′-(trimethylammonio)propyl] dodecanamide dimethylsulphate	Poly(sodium 4-styrenesulfonate) (PSS)	Oil Red O; IR-786; IR-780	Surface tension; morphology; stability; drug release studies	[94]

## 4. High Performance as Fine Chemicals

### 4.1. Magnetic Surfactants

The structure of magnetic surfactants is typical for these classes of amphipatic derivatives, with the additional presence of a magnetic moiety coming from a high-spin transition metal or lanthanide cations. The magnetic part can comprise the surfactant’s counterion or a fragment of the surfactant’s structure [5,117,118]. Accordingly, magnetic surfactants can be anionic, cationic or nonionic. There are some interesting reports in the literature, mainly on single-head–single-tail magnetic surfactants [5]. The aqueous solutions of such surfactants are paramagnetic, attaining their magnetic properties in a magnetic field. Magnetic surfactants have several advantages over nonmagnetic and magnetic nanoparticles (MNPs). Compared to MNPs, one can indicate the following advantages: facile synthesis, better biocompatibility, good aqueous stability in solution, enhanced dispersibility and increased effective binding. They can be useful in fabricating materials for selected applications, such as catalysis, drug delivery, water treatment, biotechnology and several oil-based industries for magnetic-driven delivery or separation.

Cationic magnetic surfactants constitute, typically, surface-active cations with quaternary ammonium nitrogen and anionic-type counterions bearing appropriate metal complexes [5,118,119]. Thus, it must be emphasized that the counterion is responsible for the magnetic properties of the surfactant. The most common metal complexes are iron, holmium, cerium, gadolinium and manganese halides (single or mixed salts with bromine or chlorine). In addition to the widespread single-headgroup–single-tail surfactants with one counterion, there are known gemini-type magnetic surfactants with two counterions and unique double-chained or triple-chained surfactants comprising bivalent (or trivalent) cations and two (or three) amphiphilic anions [5,120]. On the other hand, didodecyldimethylammonium cations may constitute an amphiphilic part of a magnetic surfactant with an iron-based counterion. Gemini-type magnetic surfactants may be easily obtained by stirring the metal trihalide with a suitable gemini surfactant in methanol: two moles of proper metal trichloride/tribromide per one mole of surfactant molecules. The main advantages of gemini-type magnetic surfactants are their superior physicochemical properties when compared with their single-tail–single-head analogues.

Some examples of multifunctional magnetic surfactants and their linear (single-chain single-headgroup) analogues are shown in Table 4. Gemini and especially double-chain single-head group magnetic surfactants exhibit at least one or two orders of magnitude lower values of CMC [5,117,118]. The characteristic feature of gemini-type magnetic surfactants is the strong dependence of their behavior in the magnetic field on the type of counterion. Iron and gadolinium salts are paramagnetic but do not exhibit saturation magnetization (even if the maximal magnetic field of 6000 Oe is introduced). In comparison, cerium salts are diamagnetic (exhibiting a linear decrease in magnetic moment with increasing magnetic field). In general, gemini-type magnetic surfactants exhibit better thermal stability when compared with gemini-type dibromides (typically, dibromides start to decompose at temperatures below 200 °C while their magnetic analogues may be stable up to around 115–250 °C) [5,117,118,120,121]. Several gemini-type magnetic surfactants comprise a group of unconventional surfactants consisting of two hydrophobic chains, three different hydrophilic groups (two quaternary ammonium salts and one hydroxylic group) and two magnetic counterions [121]. A unique group of magnetic surfactants are compounds with metals (e.g., manganese or gadolinium) complexed with the correct number of nitrogen atoms containing ring-type structures [122,123]. Such groups of surfactants exhibit easy tunability in terms of their surface tension and wettability due to anti-Curie behavior. They also may form liquid crystal phases.

Multifunctional magnetic surfactants constitute a new group of novel specialty surfactants that have not yet been widely studied. The described unique features show their exceptional performance properties, especially in the field of novel stimuli-responsive materials.

### 4.2. Capping Agents

Capping agents, or sometimes so-called capping ligands, comprise “binding molecules” and interfacial stabilizers that are utilized in relatively trace amounts during the preparation of metal-capped nanoparticles. Various cationic surfactants, frequently used polymers (PVP, PEG and PGA), as well as thiols (dodecanethiol and thioglycerol), are used as chemical capping agents in the vast majority of metal nanoparticle manufacturing techniques [125,126,127]. These molecules primarily inhibit the fabricated nanoparticles’ over-growth and increase the reduction kinetics on the nanoparticles’ surfaces by building complex structures with the metal ions from the precursor salts. Therefore, all surface-modifying compounds alter the surface chemistry, shape and size distribution of the fabricated nanoparticles because the variety in their unique structural features are attributed to the stabilizing action on their surface [128,129,130,131,132,133]. The controlled nanoparticles’ surface composition, as well as their structural and morphological features acquired via the capping processes are crucial in terms of evaluating the vital usages of nanoparticles and their interactions with biological components under the circumstances of alleviating cellular toxicity. Therefore, in order to be effectively utilized within the biological system, capping representatives are required to be non-toxic, biodegradable, biocompatible, easily distributed and biosoluble. As a result, their non-specific association with biological components is reduced, resulting in a reduction in the toxicity that they might cause to cells [133,134,135]. Nanoparticles made of noble metals, such as gold (AuNPs) and silver (AgNPs), have been put to extensive use in biomedicine because of their distinctive physicochemical properties. Bimetallic nanoparticles are those composed of two different metals, such as FeCo, FeNi and FePt NPs, which include iron and display magnetic and superparamagnetic characteristics with potential use in medical diagnostic imaging and drug administration [136,137,138].

It is highly encouraged that current research in the area of surfactants also focuses on synthesizing new surfactants that can potentially address the challenges associated with multicharge surfactants. Some scarce and interesting reports on the application of cationic multiheaded surfactants as efficient capping and stabilizing agents have been evaluated, and these largely concern the fabrication of AgNP or AuNP nanoparticles, demonstrating a wide range of technological capacities, particularly in biomedical applications, such as antimicrobials, drug nanocarriers, functional coatings, diagnostics probes and optoelectronic platforms [139]. AgNPs tend to agglomerate due to their high surface energy. The capping agents eliminate the uncontrolled growth due to the agglomeration of nanoparticles by forming a protective layer. Thus, they control the size and morphology of the nanoparticles and influence nanostructure stability. The use of multicharge surfactants as capping agents for AgNP has many advantages. The positively charged head groups may lead to stronger interactions with the nanoparticles; the hydrophobic chain length could form a satisfactory steric hindrance around the nanoparticles and, hence, can protect the nanoparticles from aggregation and act as a better stabilizer [4]. Due to their high hydrophobicity and morphology control, gemini surfactants have been considered one of the best shape-directing agents over classical single-tail–single-head cationics [4,140]. Capped AgNPs have been successfully evaluated in regard to their antimicrobial activity in the function of multicharge surfactant-based capping agents in order to comply with safety requirements [139]. One of the practical utilizations of cationic gemini-type surfactants in the stabilization of AgNPs was presented by S. He et al. [141]. The research proved that dimethylene-1,2-bis(dodecyl dimethylammonium bromide; 12-2-12) used in the synthesis of high-concentration silver nanoparticles resulted in noticeable long-term stability and particle sizes lower than 15 nm with an especially narrow size distribution. The conclusion presented by the authors revealed that the gemini-type surfactants exhibit a more stable and efficient capping ability in stabilizing AgNPs than conventional single-tail–single-head agents due to their double-head architecture and high charge density [141]. Another example worth noting in the preparation of nanoparticles is the employment of trimeric cationic surfactants in the synthesis of AuNPs. Wang et al. [19] described the high efficiency of tri(dodecyldimethylammonioacetoxy)diethyl-triamine trichloride (DTAD) in the formation of gold nanocrystals because of its strong capacity for adsorption at the interface. The remarkable efficacy of DTAD may be attributed to the strong electrostatic connection between the multi-charged head groups and Au facets, the hydrogen bonding across the charged head groups and the greater hydrophobic attraction of hydrocarbon chains. On the other hand, two research groups reported studies concerning the utilization of gemini-type surface agents in the formation and stabilization of surfactant-capped gold nanoparticles. M. Pisárčik et al. [142] synthesized a wide group of cationic gemini surfactants made of two ammonium headgroups, two dodecyl alkyl tails, and a -CH_2(n)_ with a variable number of carbon atoms, i.e., n = 2, 4, 6, 8, 10 and 12 (alkanediyl-α,ω-bis(dimethyldodecylammonium bromides). It has been shown that the creation of a stable nanodispersion is facilitated by the inclusion of two hydrophobic moieties and two positive charges inside a single cationic gemini molecule. Therefore, M. Pisárčik et al. [142] decided to analyze the correlation between spacer length in gemini structures and the stabilization of AuNPs. Their DLS studies of mean particle size revealed that the diameter of gold nanoparticles stabilized with short spacer gemini surfactants was greater than that of AuNPs stabilized with medium-length spacer gemini surfactants. In the case of the developed Au/12-2-12 and Au/12-4-12 nanoparticles, there was a significant increase in hydrodynamic size, which was associated with micellization. However, AuNPs coated with a medium/long spacer gemini molecule were 10–15 nm smaller than those coated with a short spacer gemini molecule (30–60 nm). Nevertheless, all synthesized cationic gemini surfactants, when capped with AuNPs, showed positive zeta potential values (from +44 to +90 mV) for all of the spacer species (n = 2–12). Meanwhile, R. M. Giráldez-Pérez et al. [143] introduced a delivery vehicle that was based on innovative gold nanosystems with gemini surfactants. The idea behind that was to offer a transport system for microRNA (miRNAs) chains as promising therapeutic targets for various diseases. These gemini surfactants offered a high capacity to induce the miRNAs’ compression, along with great stability and the ability to enter target tissues and cells. They performed a synthesis of AuNPs with positive surface charge capped with 16-Ph-16 (*N*,*N*′-[1,3-phenylene-bis(methylene)bis[*N*,*N*′-dimethyl-N-(1-hexadecyl)]-ammonium dibromide) and 16-3-16 (*N*,*N*′-bis(dimethylhexadecyl)-1,3-alkanediammonium dibromide) gemini structures. Finally, the nanoparticles were covered with miR-21 polymer, forming Au@16-Ph-16/miR-21 and Au@16–3-16/miR-21 systems, respectively. Nanoparticles were studied by measuring their size and charge distribution using zeta potential, transmission electron microscopy, atomic force microscopy, dynamic light scattering and UV–Vis spectroscopy. Moreover, in vivo toxicity studies were also assessed. Based on the results of the tests, the positive zeta potential values of the nanosystems exhibited values from 31 to 50 mV for the Au@16-3-16 derivatives and between 35 and 67 mV for the Au@16-3-16 derivatives. The zeta potential values that were observed, along with the single peak of zeta potential for each formulation, showed that all of the systems were stable in terms of flocculation and aggregation. The findings from the DLS demonstrate that taking into account the fact that the size of the precursor Au@16-3-16 was less than the size that corresponds to Au@16-Ph-16, the size of the Au@16-Ph-16/miR-21-covered nanosystems was only slightly bigger than that of the Au@16-3-16/miR-21 derivative, and this was the case regardless of the concentration of miR-21. This might be because the phenyl rings of the Au@16-Ph-16 are able to enter the RNA base pairs via partial intercalation, which results in the backbone of the biomolecule being stretched and distorted structurally. Finally, R. M. Giráldez-Pérez et al. [143] came to the conclusion that the gemini surfactants, 16-3-16 and 16-Ph-16, had great potential in terms of inducing miRNA compression while also being neutral due to their low toxicity. These surfactants were first developed with the intention of functioning as prospective detergents, and as a result, these molecules have the potential to be considered allergenic to fatty tissue. As a result of all of this, the number of miRNAs that are required to function on the target tissue might be significantly reduced.

### 4.3. Biocidal Agents

Generally, cationic surfactants exhibit significant antimicrobial activity (i.e., activity against Gram-positive and Gram-negative bacteria and fungi), and they can be used as active substances in a variety of biocidal formulations. They also possess antifouling activity in coating abiotic surfaces (e.g., plastic, stainless steel) and protect endoprostheses and implants from the adhesion of microorganisms as well as inhibit biofilm formation, which is extremely important in terms of medical applications. However, the widespread usage of surfactants has resulted in the development of disinfectant resistance among microorganisms. Therefore, there is a need to synthesize new compounds to which microorganisms do not show resistance. In the literature, there are examples of various series of alkylammonium-type multiheaded surfactants with biological activities against different microorganisms and the relationship between their chemical structure (length of alkyl chains, linker, counterion, number of hydrophilic head groups) and the antimicrobial activity is widely described [7,144,145]. The surfactant activity and ability to reduce filamentation, as well as its adhesion to polystyrene, glass, stainless steel and silicone, were analyzed against the selected Gram-positive and Gram-negative strains as well as fungi [144,146,147]. Multiheaded cationic surfactants (MHCSs) are extensively studied as biocidal agents due to their ability to disrupt bacterial membranes and annihilate microorganisms [148]. These specific cationic surfactants contain multiple positively charged head groups and a hydrophobic tail, which enables them to interact with and disrupt the lipid bilayer of microbial membranes. The biocidal activity of MHCSs can be attributed to their ability to penetrate the cell membrane leading to cell death. The MHCSs interact with microbial membranes by electrostatic attraction between their positively charged head groups and the negatively charged microbial membrane. The hydrophobic tail of the MHCS inserts into the lipid bilayer, leading to membrane disruption and the loss of membrane integrity. This can result in the leakage of intracellular contents, the loss of cellular energy, and ultimately, cell death. Current research shows that the mechanism of action of MHCS is similar to that of other cationic surfactants, such as quaternary ammonium compounds (QACs) [149]. The biocidal activity of multiheaded cationic surfactants is dependent on the number of positively charged groups in the surfactant molecule, as well as the multiplicity, length and nature of the hydrophobic tails.

Recent research investigations have demonstrated that in contrast to quaternary ammonium salts such as benzalkonium chloride (BAC), which have been used for decades, multicationic disinfectants are more effective and rarely lead to the development of resistance by various types of microorganisms [150,151]. The weaknesses of traditional quaternary ammonium disinfectants have become more apparent in the last few decades, so many research teams are working on the development of novel and more effective MHCS structures. Haldar et al. [7,44] were one of the first research teams that described the synthesis and antibacterial activity of multiheaded dicephalic cationic surfactants. These scientists used moieties composed mainly of trimethylammonia or pyridine as cationic groups. In addition, the multiplied cationic head groups were covalently linked via scissile ester-type linkages. The presence of a cleavable ester group that undergoes spontaneous hydrolysis under physiological conditions, as demonstrated by Halder’s team, allows these active substances to readily biodegrade. Moreover, they found that dicephalic MHCSs with triple-head groups (trimethylammonia (T3) or pyridine (P3)) were the most active of all synthesized substances, respectively: the minimum bactericidal concentrations (MBCs) for T3 were less than 11.3 uM and for P3 were less than 6.5 uM, while the single-headed reference surfactants showed a very weak killing effect on bacteria: MBCs for cetyltrimethylammonium bromide (CTAB) were not less than 16.9 uM and for cetylpyridinium bromide (CPB) were 10.4 uM, respectively. Therefore, the Halder research team’s efforts to produce dicephalic cationic surfactants with an easily cleavable ester bond in their structure may eventually result in more effective disinfectants and antiseptics for food and body surfaces than conventional single-headed cationic surfactants. Star-shaped surfactants with multiheaded groups are part of another significant subgroup of MHCSs [152,153,154,155]. The multi-cationic homologs of benzalkonium chloride (BAC) are one of the structures that research teams are now working on, and they look the most promising. In the paper published by Toles et al. [150], the researchers successfully obtained triscation BAC analogues (Tris-BAC) and showed that their antimicrobial activity was significantly greater than that of monocationic BAC. In this study, the authors demonstrated that multicationic derivatives of BAC had biological activity against the Gram-positive and Gram-negative bacteria in the single-digit micromolar range. They also examined that such derivatives had MIC values from two to thirty-two times better than BAC. In particular, Toles et al. remark the improvement in the efficiency of MHCS against some of the most worrisome bacteria that are currently circulating in the public space, such as *Acinetobacter baumannii* and *Pseudomonas aeruginosa*, which are exhibiting an increasing level of resistance to standard disinfectants. It is a generally held belief that Gram-negative bacteria, with the barrier function of the outer membrane, are more difficult to eradicate than Gram-positive bacteria [144,156,157]. Furthermore, it is estimated that approximately half of all illnesses are caused by Gram-negative *Escherichia coli* [158]. In the research reported by Zhou et al. [144] the scientists obtained trimeric (DTAD), Ref. [159] tetrameric (PATC) [160] and hexameric (PAHB) [161] star-shaped MHCS structures in which hydrophobic chains and charged hydrophilic head moieties were connected by an amide-type spacer group. Zhou et al. claimed that the gained structures were very effective against Gram-negative *E. coli*, with a minimum inhibitory concentration (MIC) of 0.93 μM to 1.70 μM and minimal toxicity to mammalian cells. They found that the antibacterial activity of the cationic oligomeric surfactants increases with the degree of oligomerization of MHCS (i.e., PAHB > PATC > DTAD). In the literature, there are also descriptions of oligomeric MHCS structures [162,163,164]. Shaban et al. [165] reported one of the most remarkable structures, which exhibits exceptionally intriguing antibacterial and antifungal activities. Through the process of alkylation of the alginate ester, they were able to obtain multicationic polymeric surfactants with several types of alkyl chains, including octyl, dodecyl and hexadecyl (respectively, denoted as ALGOB, ALGDB and ALGHB). The produced polymeric surfactants exhibited good efficacy against fungi such as *Candida albicans* and *Aspergillus niger*, as well as bacteria such as *Bacillus subtilis* (G+), *Staphylococcus aureus* (G+), *Escherichia coli* (G−) and *Pseudomonas aeruginosa* (G−). In this research, Shaban et al. also described additional significant properties of the fabricated MHCS structures that make them able to also be used as corrosion inhibitors for mild carbon steel when subjected to an aggressive acidic environment.

However, it is important to note that the antibacterial and antibiofilm efficacy of multiheaded cationic surfactants can be influenced by various factors, such as the concentration of the surfactant, pH, temperature and the presence of organic matter. In addition, the use of these surfactants as antifouling agents may have unintended consequences, including the development of resistance in target bacteria or the disruption of beneficial microbial populations. Therefore, further research is needed to fully understand the potential risks and benefits of using MHCSs as antibacterial and antibiofilm agents. Moreover, regulatory frameworks should be put in place to ensure the safe and responsible usage of these surfactants in various applications as antimicrobial species.

## 5. Conclusions and Future Trends

This comprehensive study has attempted to review recent works on a profound class of cationic surfactants, i.e., multihead structures, concerning basic research on their self-assembly at interfaces and in solution and their various technologically appreciable chemical and biological functions. Due to the structural features of a variety of multicharge surfactants, various synthesized (including those from our group) multiheaded structures show unique functionality in relation to many specialized products (i.e., so-called fine chemicals) [1,6,7,14,46,146,166,167]. For such applications, the usage of multifunctional surfactants of high quality and purity is required; therefore, step-by-step synthetic strategies and coupling approaches are mostly used, as they may enable reactions to be conducted under mild conditions and provide easy purification steps. In the literature, much attention has been paid to the solubilization ability of multifunctional cationic surfactants and their potential use as components of new drug components, constituting a kind of matrix in the preparation of various types of nanostructures, including drug nanocarriers or templates in the synthesis of nanoscale-type materials. Accordingly, they might exhibit high biological activity toward a wide range of bacteria, viruses, fungi or algae; therefore, they can be used in disinfectants or biocides. All efforts to fabricate such new complex structures of ionic multicharged surfactants and the need to understand their behavior at interfaces is currently the subject of dynamically developing research. However, regarding the properties and performance of many customized multihead cationic structures, especially those containing dendronic head groups, only a limited number of reports are present in the literature. Systematic and extensive research is highly welcomed, such as research on new multicharge surfactant structure designs, as well as on the elaboration of convenient synthetic routes or less costly synthesis methods and understanding their self-assembly at interfaces and in solution. The use of molecular dynamics methods combined with an approach based on the thermodynamic models of multicharge surfactant adsorption is desirable because it may explain certain experimentally observed phenomena and provide the basis for developing novel materials designated for various applications.

## Figures and Tables

**Table 1:** Examples of modular synthesis based on step-by-step reactions.

No.	Starting Block	Building Block (ii Stage)	Conditions (ii Stage)	Modification (iii Stage)	Conditions (iii Stage)	Final Treatment	Ref.
1	Octadecanol	2,2,5-trimethyl-1,3-dioxane-5-carboxylic anhydride	Solvent: water:DCM mixture; catalyst: DMAP; 24 h; room temperature	Hydrolysis of cyclic acetale	Solvent: methanol; Dowex H+ resin; 24 h; room temperature	Reaction with succinic anhydride; basification with NaOH	[22]
2	Hexadecylamine	methyl acrylate (three moles per one mole of amine groups)	Solvent: methanol; 40 °C; 90 h; solvent and excess of methyl acrylate removal by distillation under reduced pressure	Formation of an amide bond via reaction of ester with ethylenedimine (20 equiv per one ester group)	Solvent: methanol; 40 °C; 80 h; solvent and excess of reagent removal by sequential azeotropic distillation	Final reaction with ethylenedimine (20 equiv per one ester group) to provide multiple amine terminal groups	[34]
3	Cyanuric chloride; tert-Octylamine	1,4-butanediamine; diisopropyl ethylamine	Solvent: THF; 100 °C; 24 h	Cyanuric chloride; diisopropylethylamine	Solvent: THF; 0–25 °C; 24 h	Reaction with Jeffamine EDR-192 and diisopropyl ethylamine in CHCl_3_; purification by gel chromatography	[36]
4	Cyanuric chloride; tert-Octylamine; 1,4-butanediamine; diisopropyl ethylamine	1,4-butanediamine; diisopropylethylamine	Solvent: THF; reflux; 24 h	Cyanuric chloride; diisopropylethylamine	Solvent: THF; 0–25 °C; overnight; reflux; overnight	Reaction with Jeffamine EDR-192 and diisopropyl ethylamine in CHCl_3_; purification by gel chromatography	[36]
5	*N*,*N*′-didodecyl ethylenediamine	Methyl acrylate (5 equiv per one amine group)	Solvent: methanol; 40 °C; 50 h; solvent and excess of reagent removal under reduced pressure	Formation of an amide bond via reaction of ester with ethylenedimine (15 equiv per one ester group)	Solvent: methanol; 40 °C; 140 h; solvent and excess of reagent removal by sequential azeotropic distillation	Final reaction with ethylenedimine (20 equiv per one ester group) to provide multiple amine terminal groups	[34]

DCM: dichloromethane; THF: tetrahydrofuran.

**Table 4:** Examples of multifunctional magnetic surfactants and their linear analogues.

No.	Surfactant Type	Structure	Synthetic Method	M_w_ [g/mol]	Cmc Value [mM]	Comments	Ref.
1	Gemini-type structure 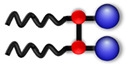	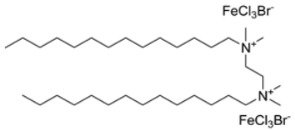	Reaction of cationic bromide (*N*,*N*,*N*,*N*′,*N*′,*N*′-Tetra-methylditetradecylethylene ammonium dibromide) with FeCl_3_ (two equiv.) in methanol; overnight, room temperature	995.181	0.106	Product obtained via solvent evaporation and drying (overnight) under reduced pressure at 80 °C Product is paramagnetic at room temperature	[118]
2		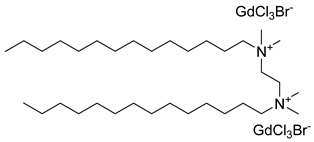	Reaction of cationic bromide (*N*,*N*,*N*,*N*′,*N*′,*N*′-Tetra-methylditetradecylethylene ammonium dibromide) with GdCl_3_ (two equiv.) in methanol; overnight, room temperature	1197.99	0.146	Product obtained via solvent evaporation and drying (overnight) under reduced pressure at 80 °C Product is paramagnetic at room temperature, weaker magnetic properties in comparison to Fe salt	[116]
3		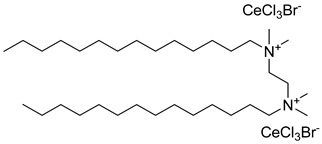	Reaction of cationic bromide (*N*,*N*,*N*,*N*′,*N*′,*N*′-Tetra-methylditetradecylethylene ammonium dibromide) with CeCl_3_ (two equiv.) in methanol; overnight, room temperature	1163.72	0.109	Product obtained via solvent evaporation and drying (overnight) under reduced pressure at 80 °C Product is diamagnetic	[116]
4		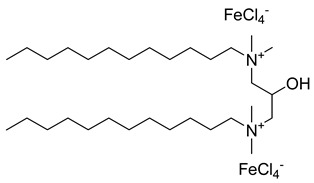	Two step reaction: coupling of *N*,*N*-dimethyldodecylamine (two moles per one mole of epichlorohydrine) with epichlorohydrone (70 °C, 8 h); followed by reaction with iron (III) chloride hexahydrate (12 h)	880.198	-	Product exhibits tunable value of surface tension (magnetic field controlled) as well as phase-inversion ability (magnetic field susceptible)	[5,121]
5	Dendronium-type structure 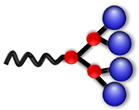	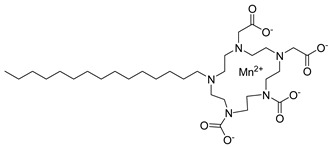	Reaction of appropriate ligand with equimolar amount of metal halide in methanol (48 h, reflux)	623.74	-	Product obtained via filtration of residues, followed by solvent evaporation under reduced pressure; product exhibit tunable surface tension and wettability due to anti-Curie behavior	[122]
6		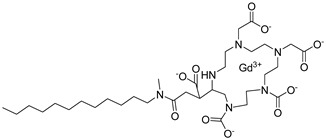	Reaction of appropriate ligand with equimolar amount of metal halide in methanol (48 h, reflux)	869.073	-	The obtained surfactants form liquid crystal phase due to differences in packing parameters when compared to typical surfactants.	[123,124]
7	Single-head–single-chain structure 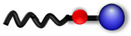	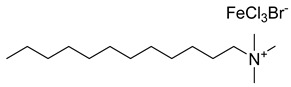	Reaction of cationic bromide (dodecyltrimethyl ammonium bromide) with FeCl_3_ in methanol; overnight, reflux	470.545	13.6	-	[5,120]
8		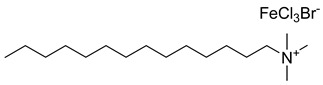	Reaction of cationic bromide (tetradecyldimethyl ammonium bromide) with FeCl_3_ in methanol; overnight, room temperature	498.598	2.46	Product obtained via solvent evaporation and drying (overnight) under reduced pressure at 80 °C	[118]
9	Single-head–double-chain structure 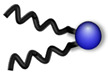	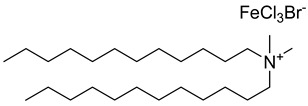	Reaction of cationic bromide (didodecyldimethyl ammonium bromide) with FeCl_3_ in methanol; overnight, reflux	624.838	0.06	Extremely low CMC value	[5,120]

## Data Availability

Not applicable.
